# Supplementary figures and images for: Quantitative evaluation of soil anti-erodibility in the fluctuation zones of rooted soil in a large reservoir, southwest of China
Source: PLoS One. 2026 Mar 10;21(3):e0336637. doi: 10.1371/journal.pone.0336637 (PMC12974866; doi:10.1371/journal.pone.0336637)

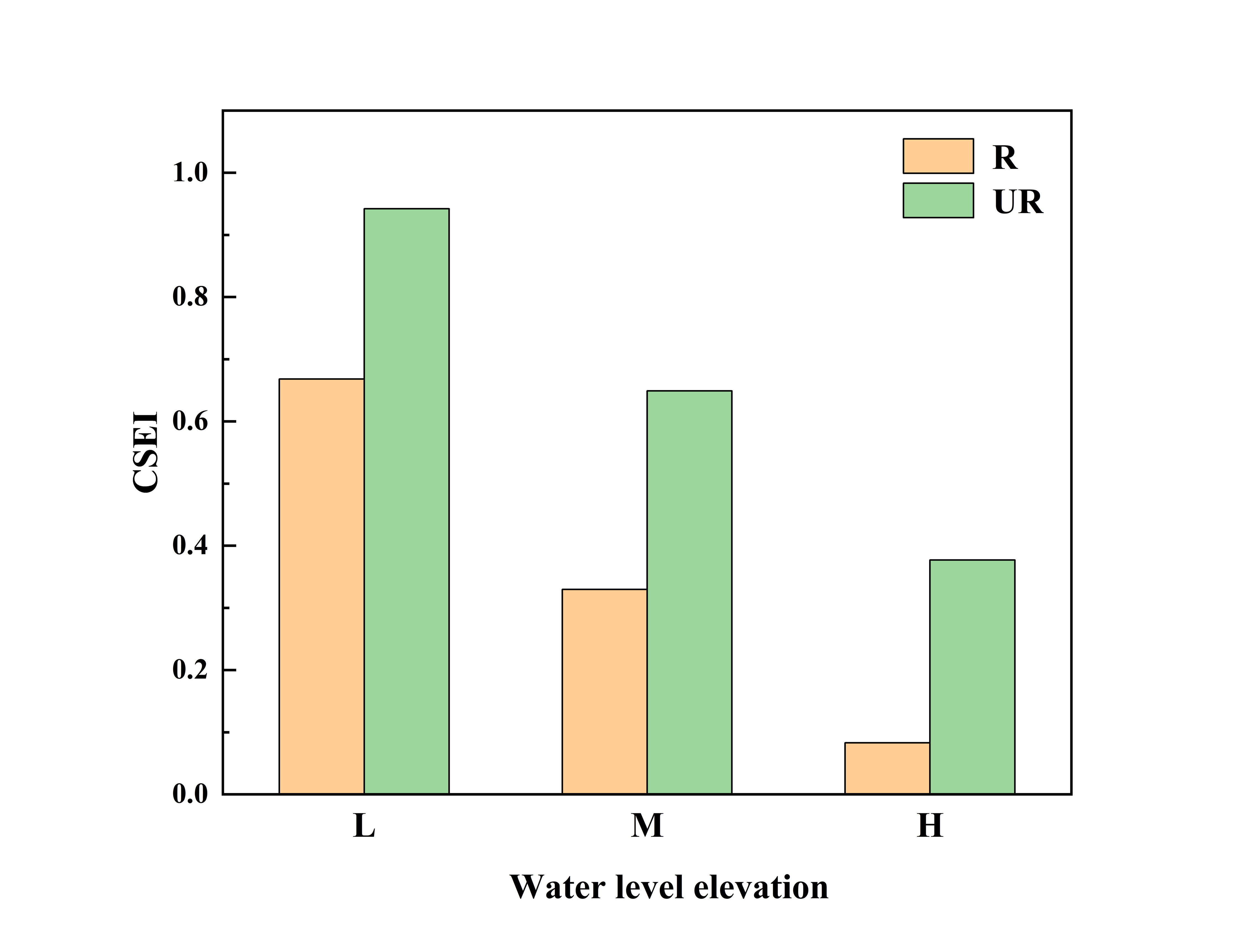

Supplement: S1 File — (ZIP) [file pone.0336637.s001.zip › S1/csei.tif]

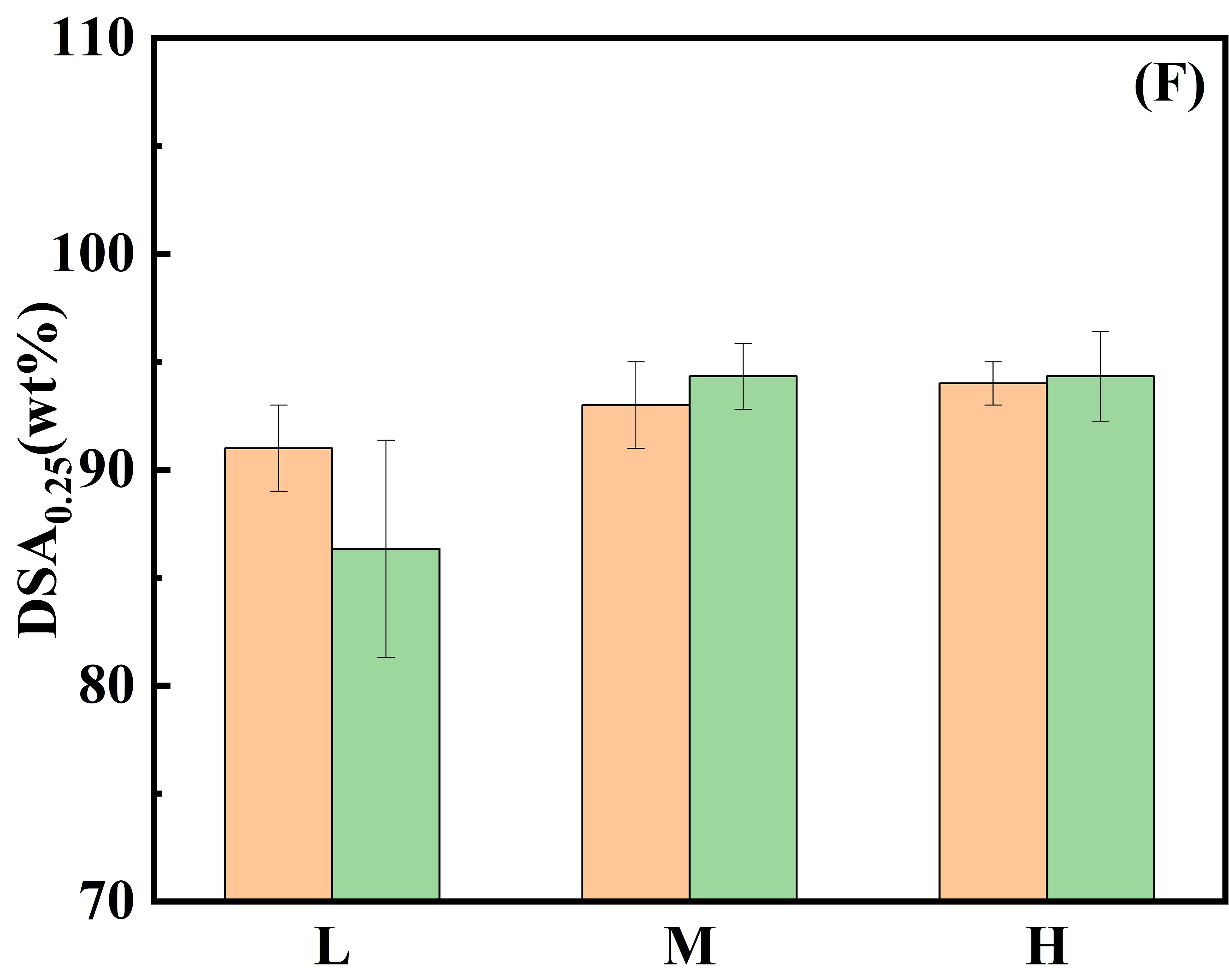

Supplement: S1 File — (ZIP) [file pone.0336637.s001.zip › S1/DSA.tif]

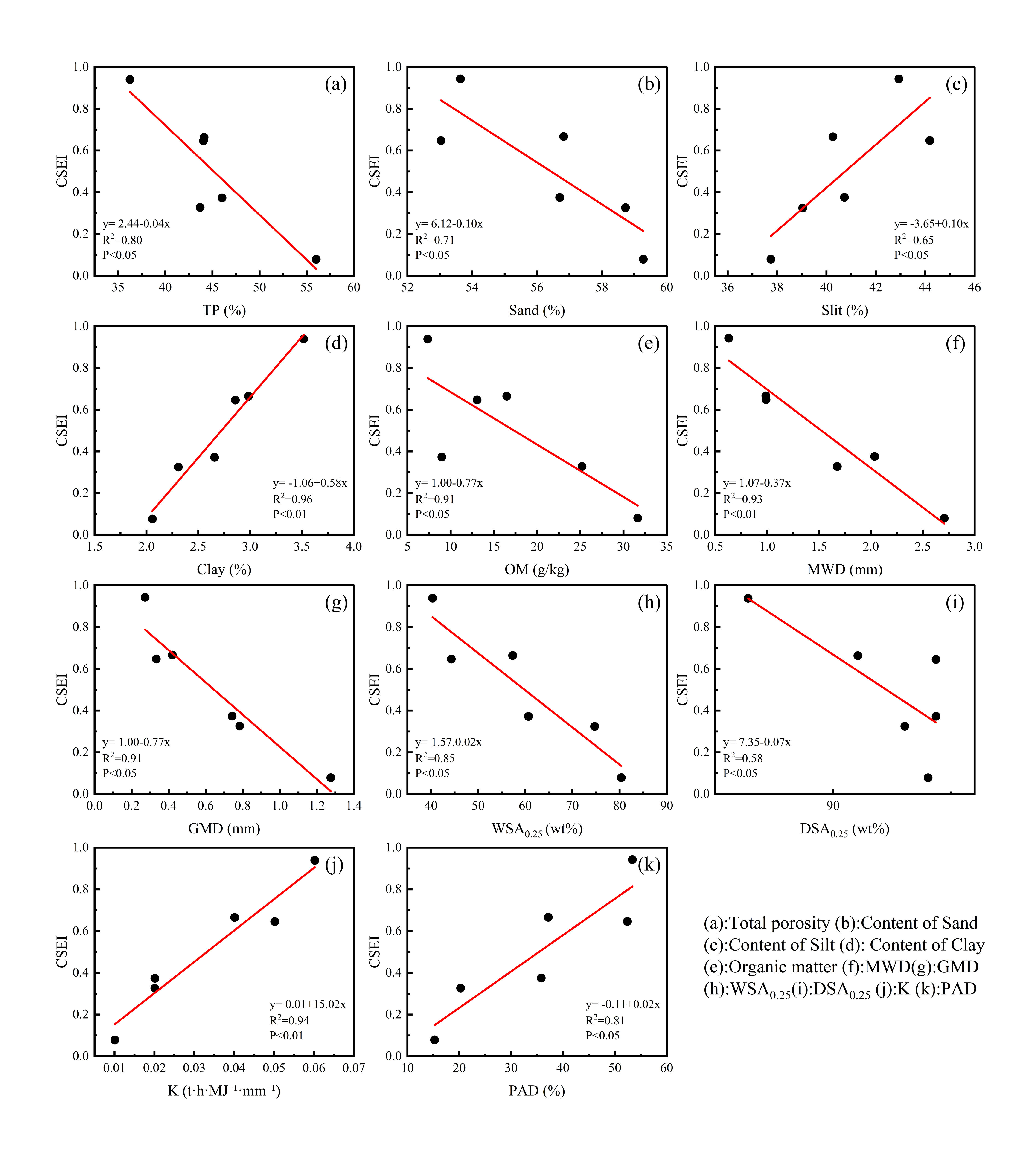

Supplement: S1 File — (ZIP) [file pone.0336637.s001.zip › S1/Fig 11.tif]

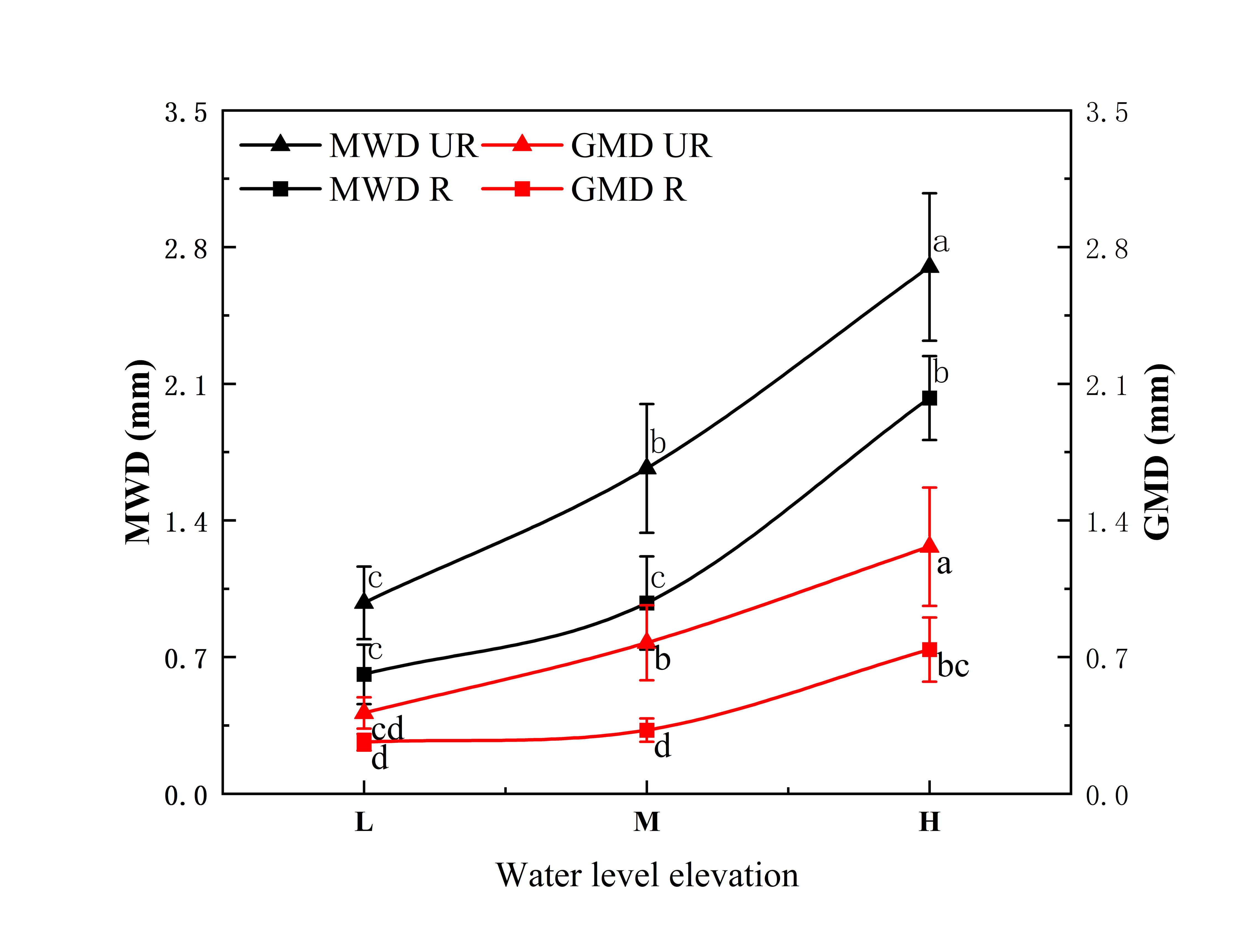

Supplement: S1 File — (ZIP) [file pone.0336637.s001.zip › S1/Fig 6.tif]

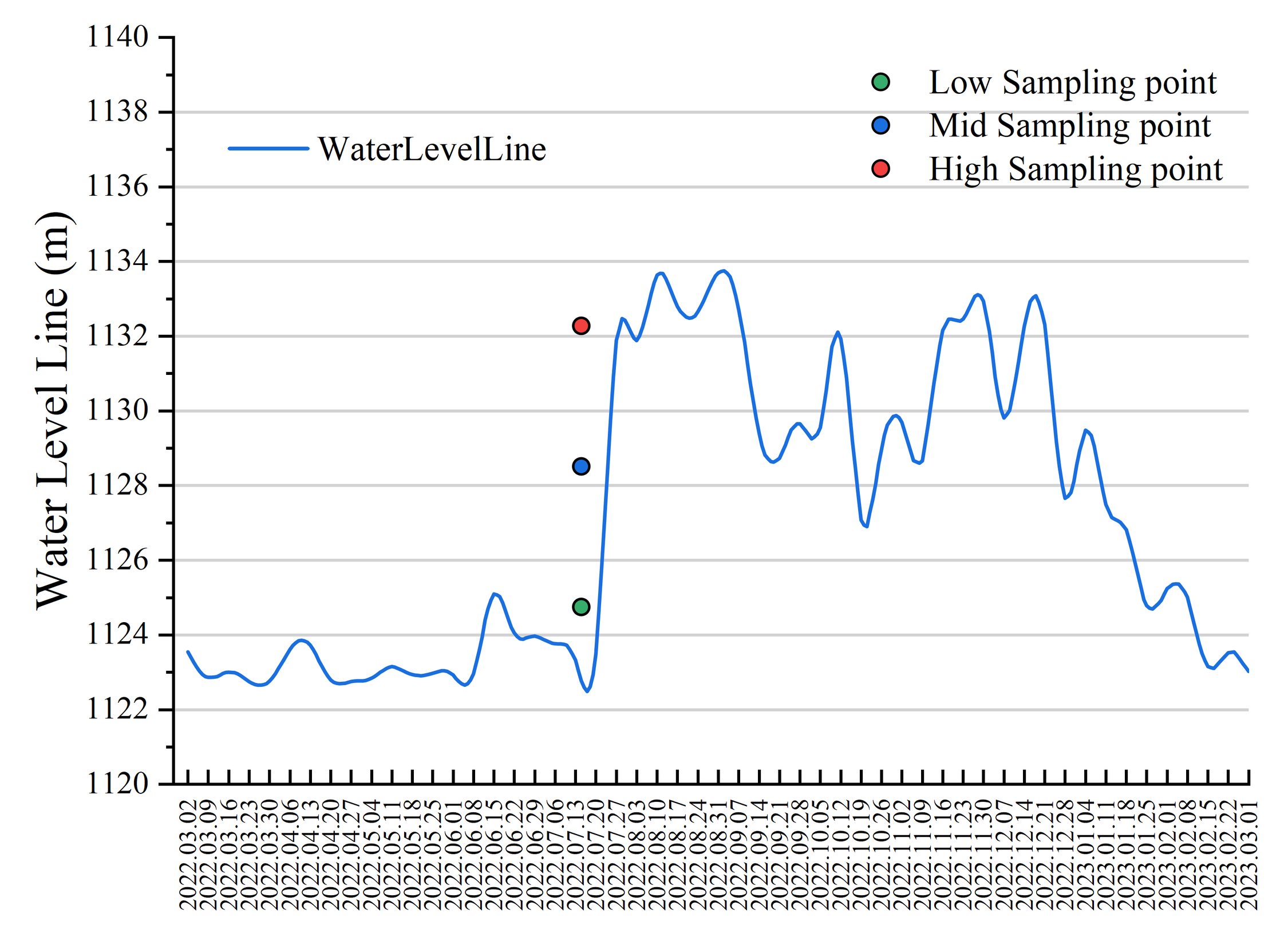

Supplement: S1 File — (ZIP) [file pone.0336637.s001.zip › S1/Fig 2.tif]

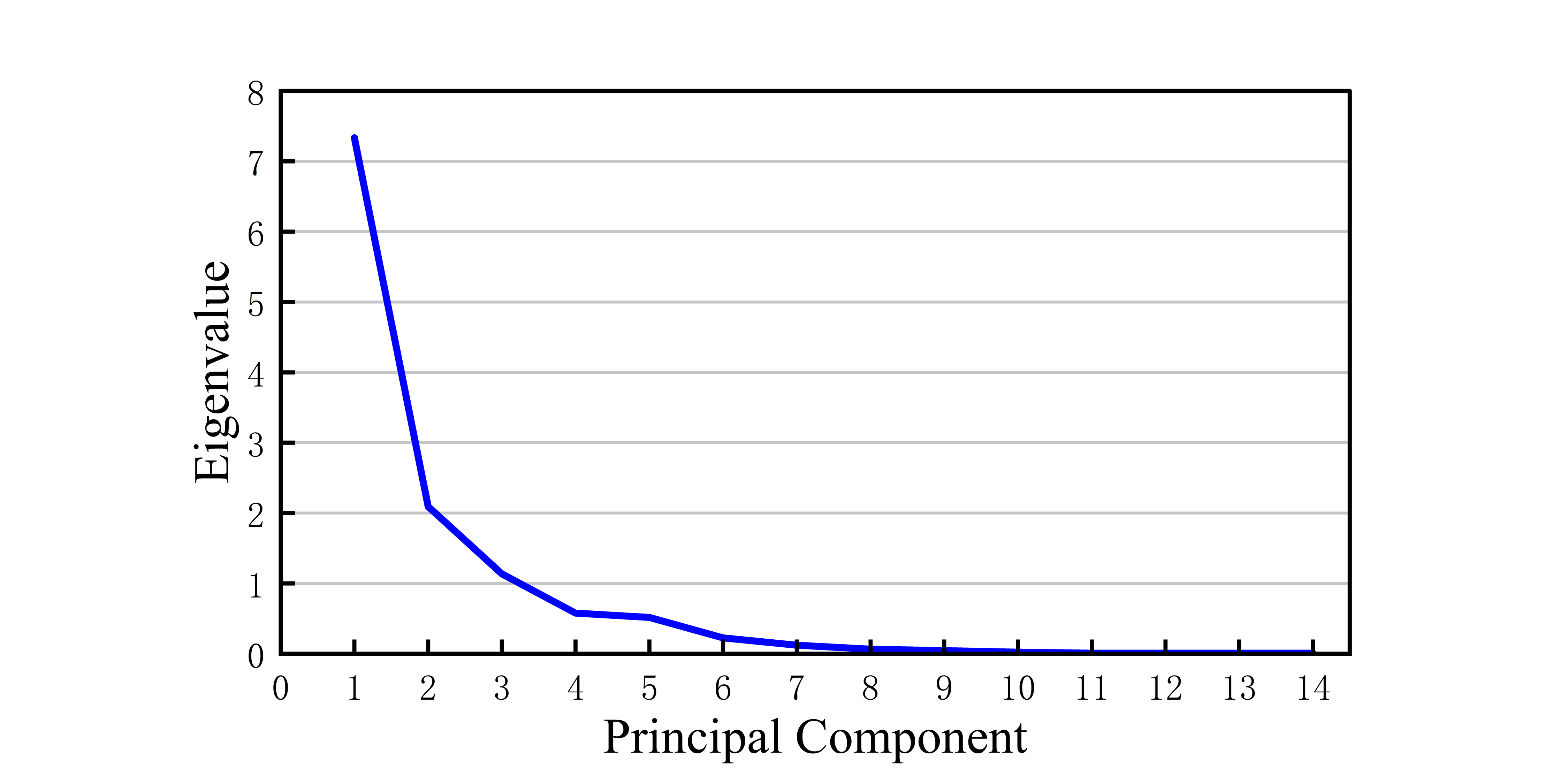

Supplement: S1 File — (ZIP) [file pone.0336637.s001.zip › S1/Fig 3.tif]

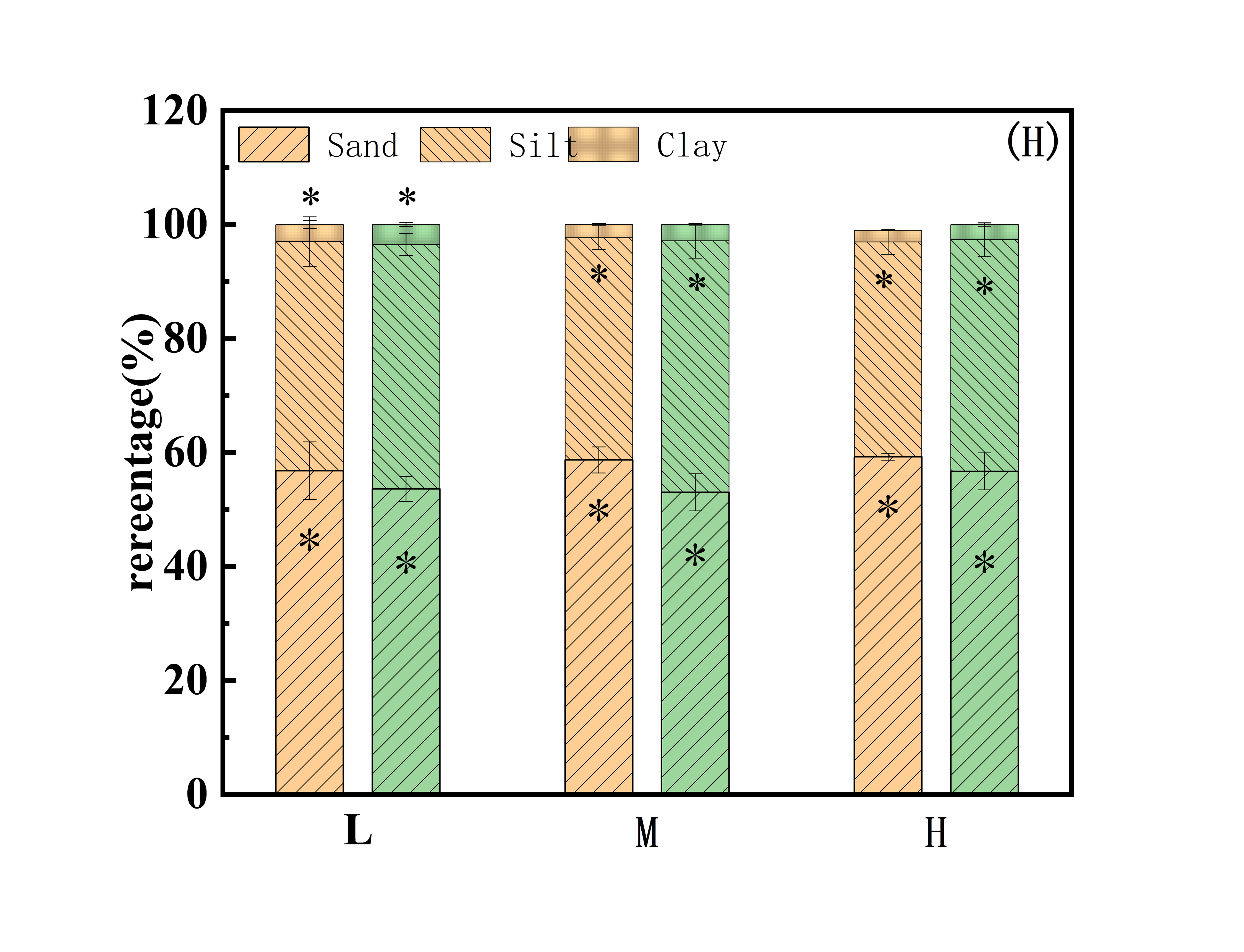

Supplement: S1 File — (ZIP) [file pone.0336637.s001.zip › S1/Fig 4 h.tif]

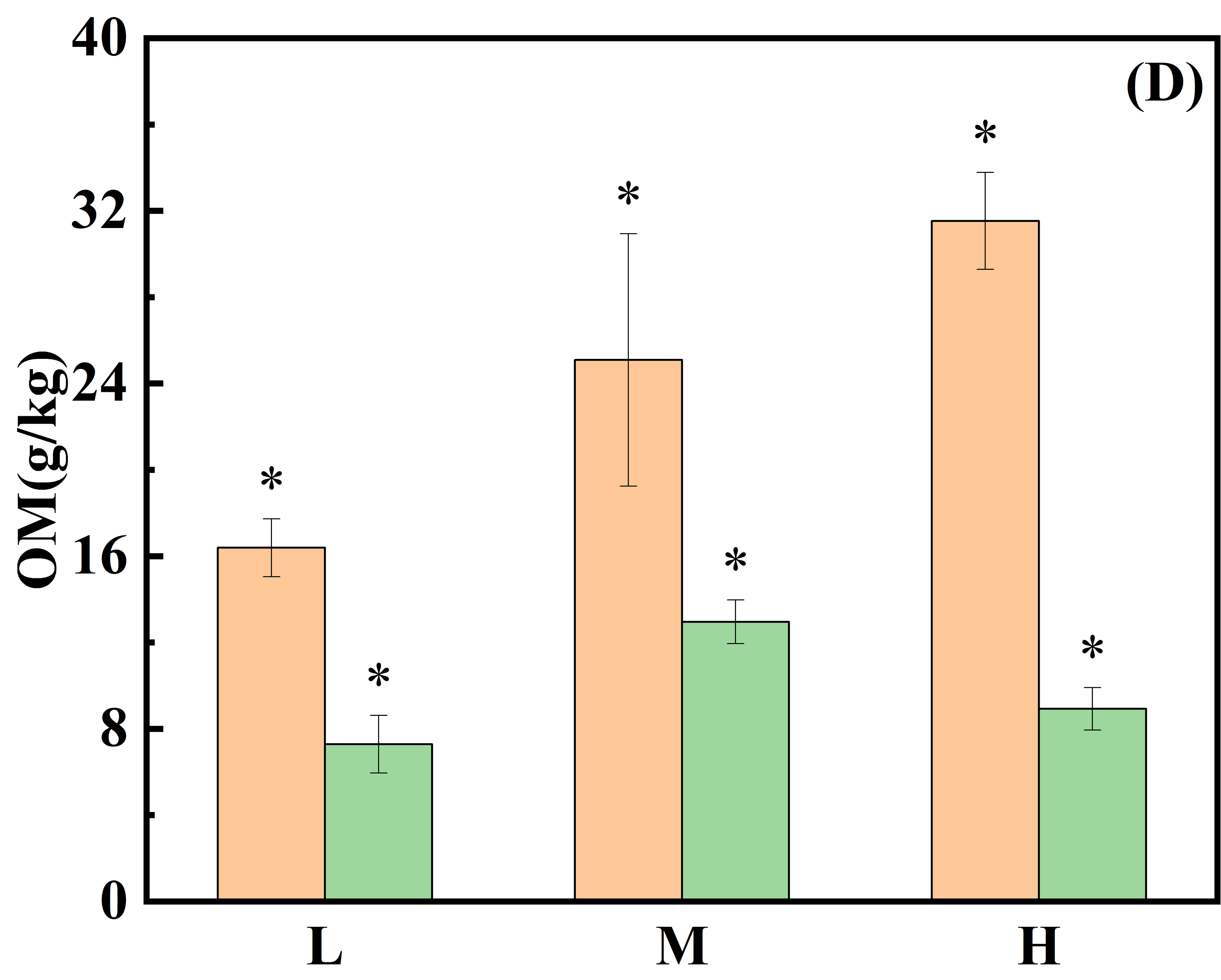

Supplement: S1 File — (ZIP) [file pone.0336637.s001.zip › S1/Fig 4 om.tif]

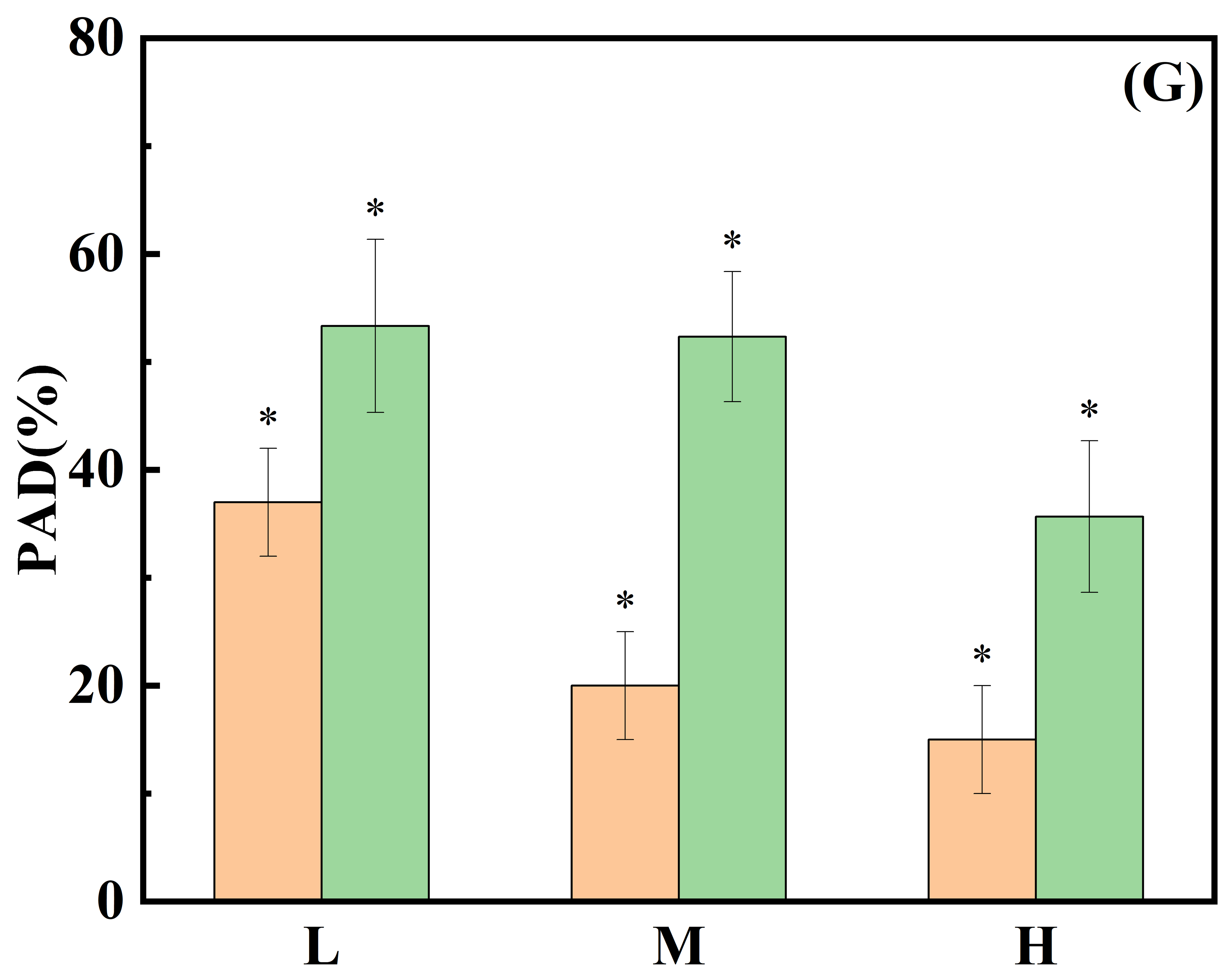

Supplement: S1 File — (ZIP) [file pone.0336637.s001.zip › S1/Fig 4 pad.tif]

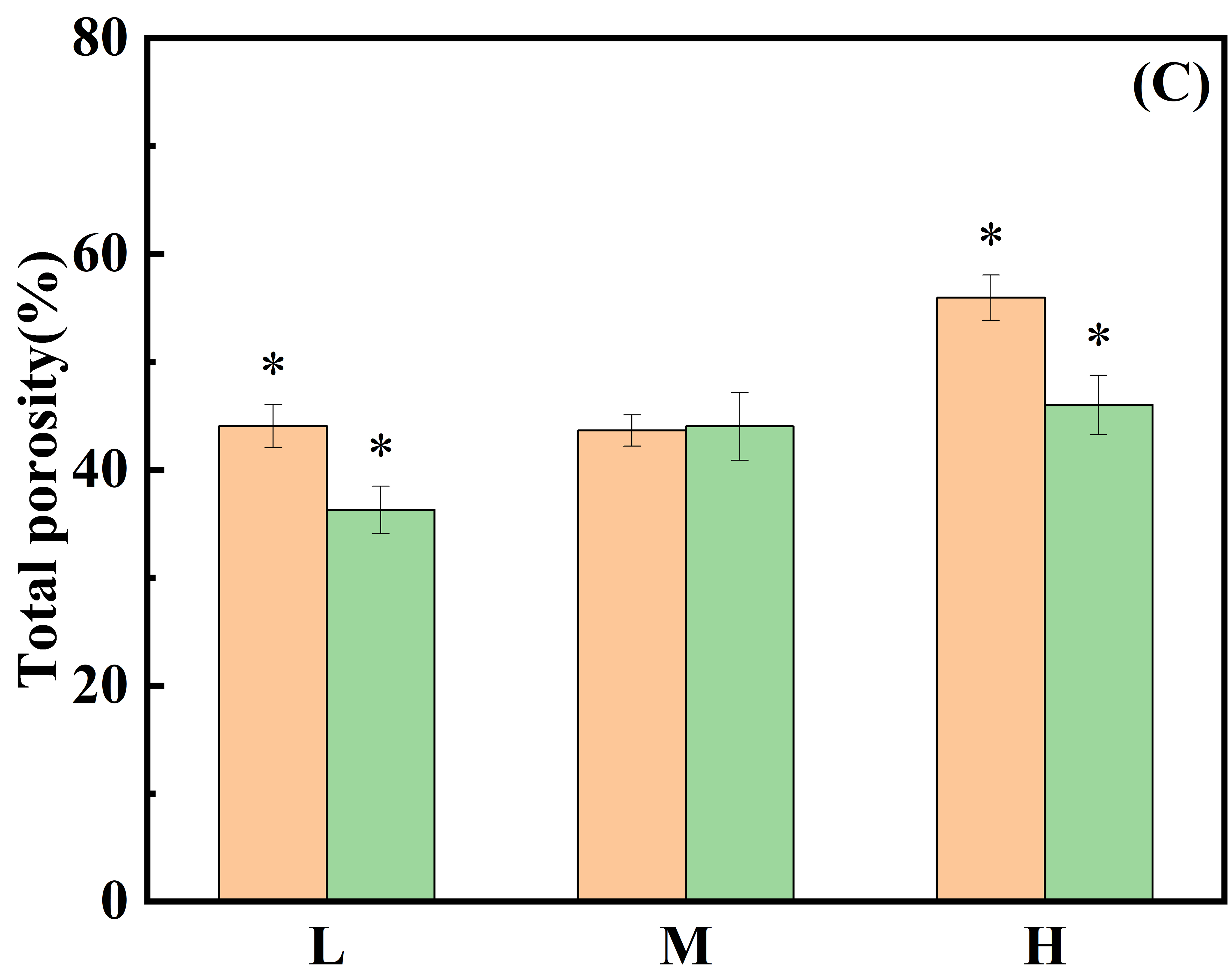

Supplement: S1 File — (ZIP) [file pone.0336637.s001.zip › S1/Fig 4 total porosity.tif]

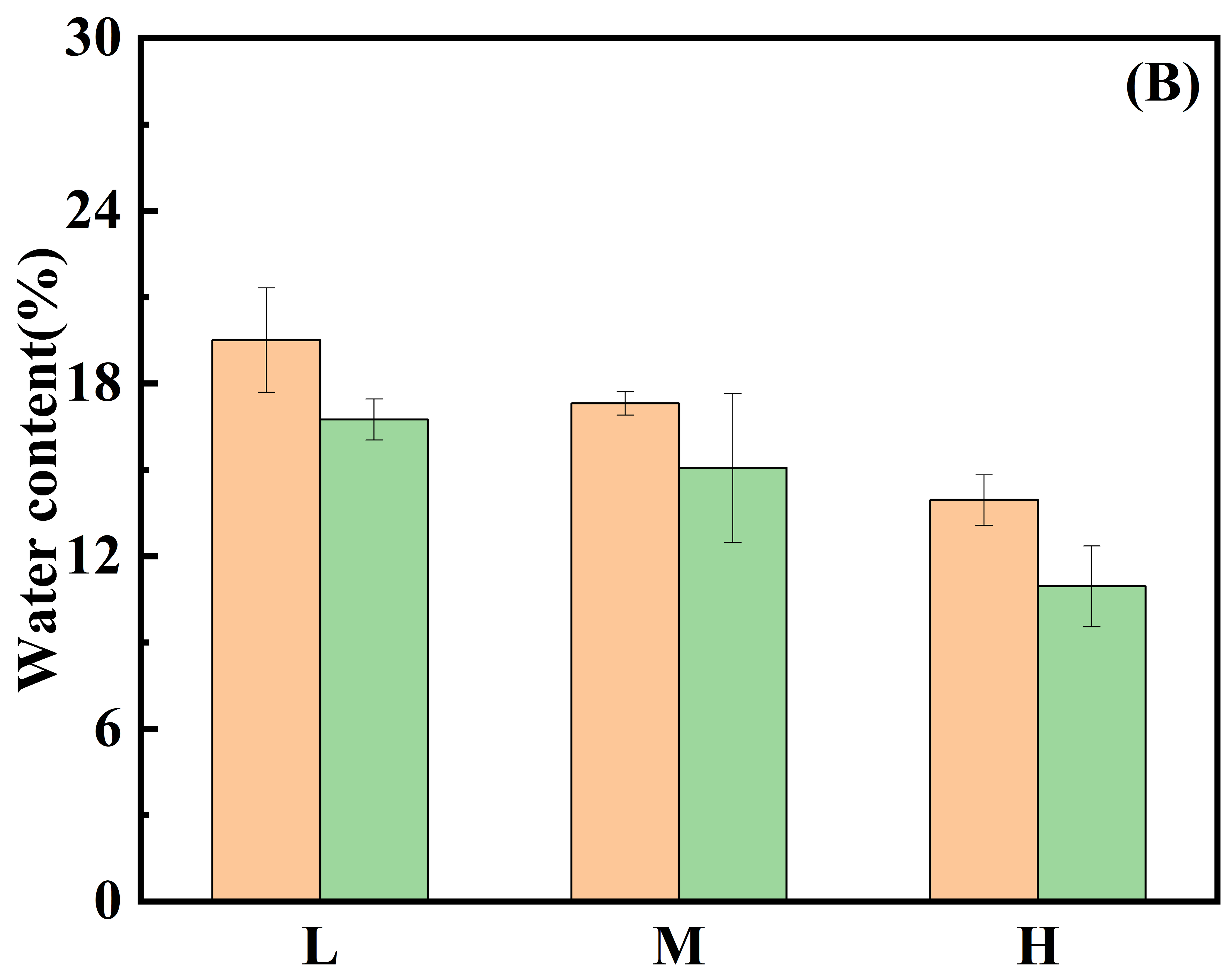

Supplement: S1 File — (ZIP) [file pone.0336637.s001.zip › S1/Fig 4 water content.tif]

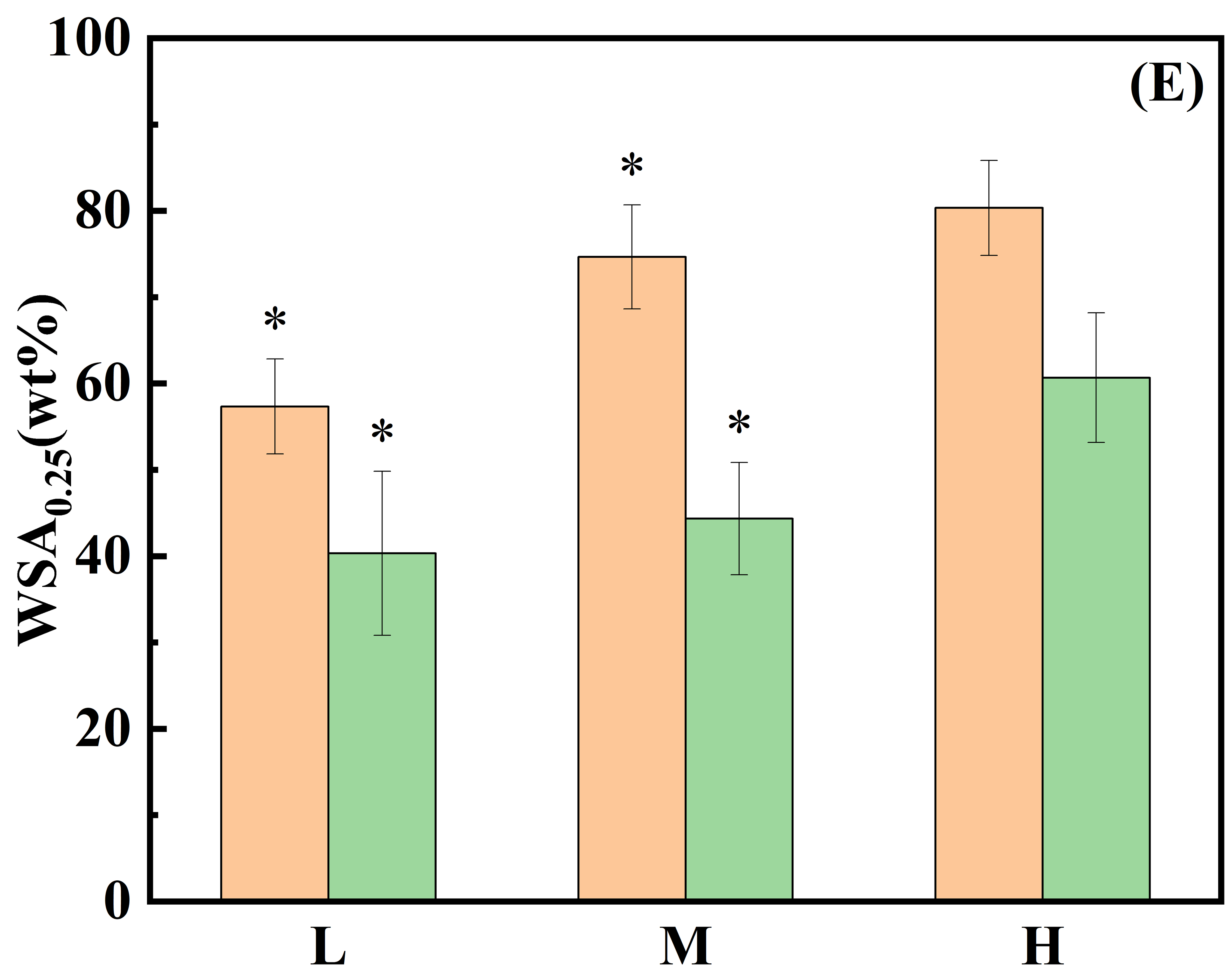

Supplement: S1 File — (ZIP) [file pone.0336637.s001.zip › S1/Fig 4 WSA.tif]

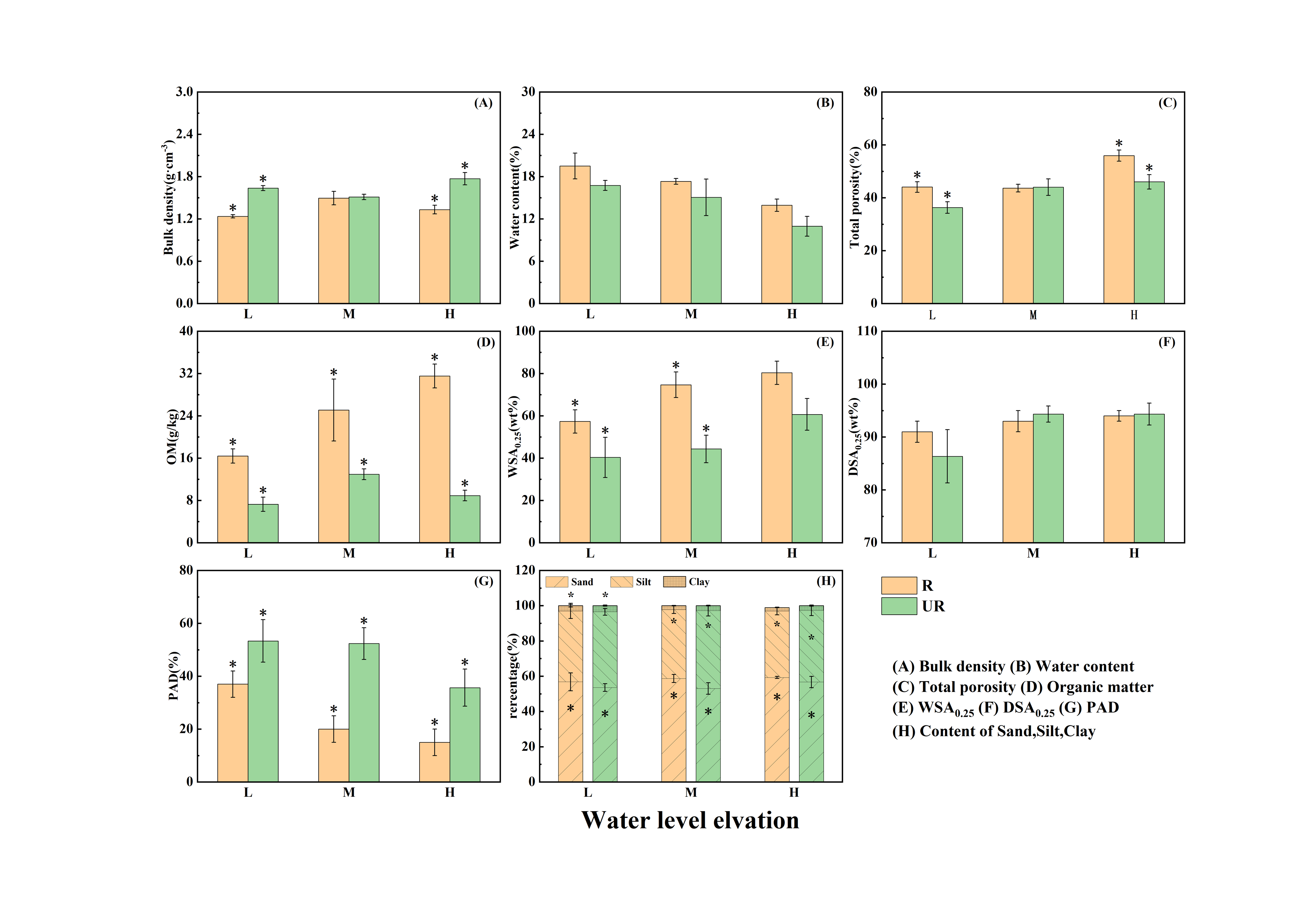

Supplement: S1 File — (ZIP) [file pone.0336637.s001.zip › S1/Fig 4.tif]

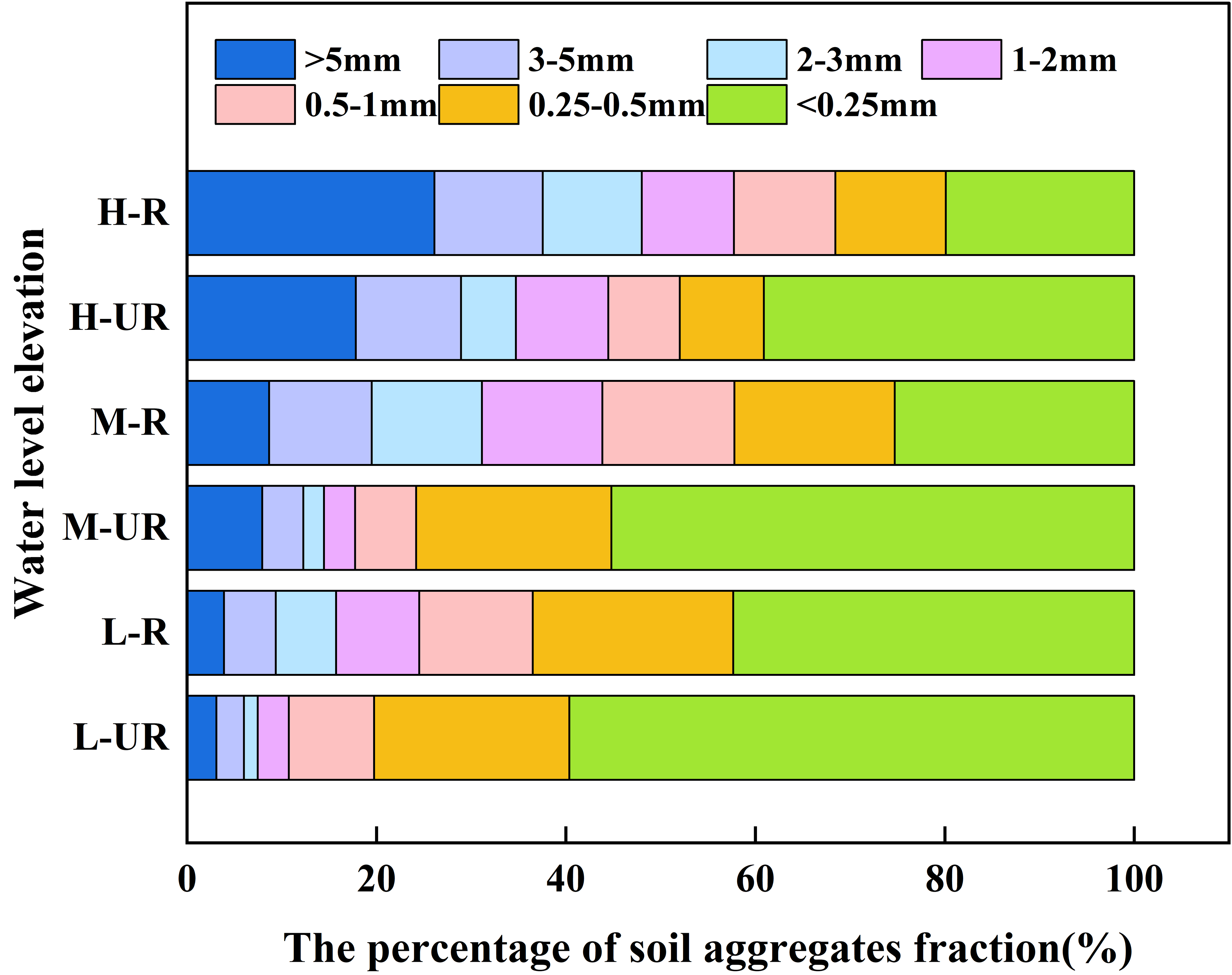

Supplement: S1 File — (ZIP) [file pone.0336637.s001.zip › S1/Fig 5.tif]

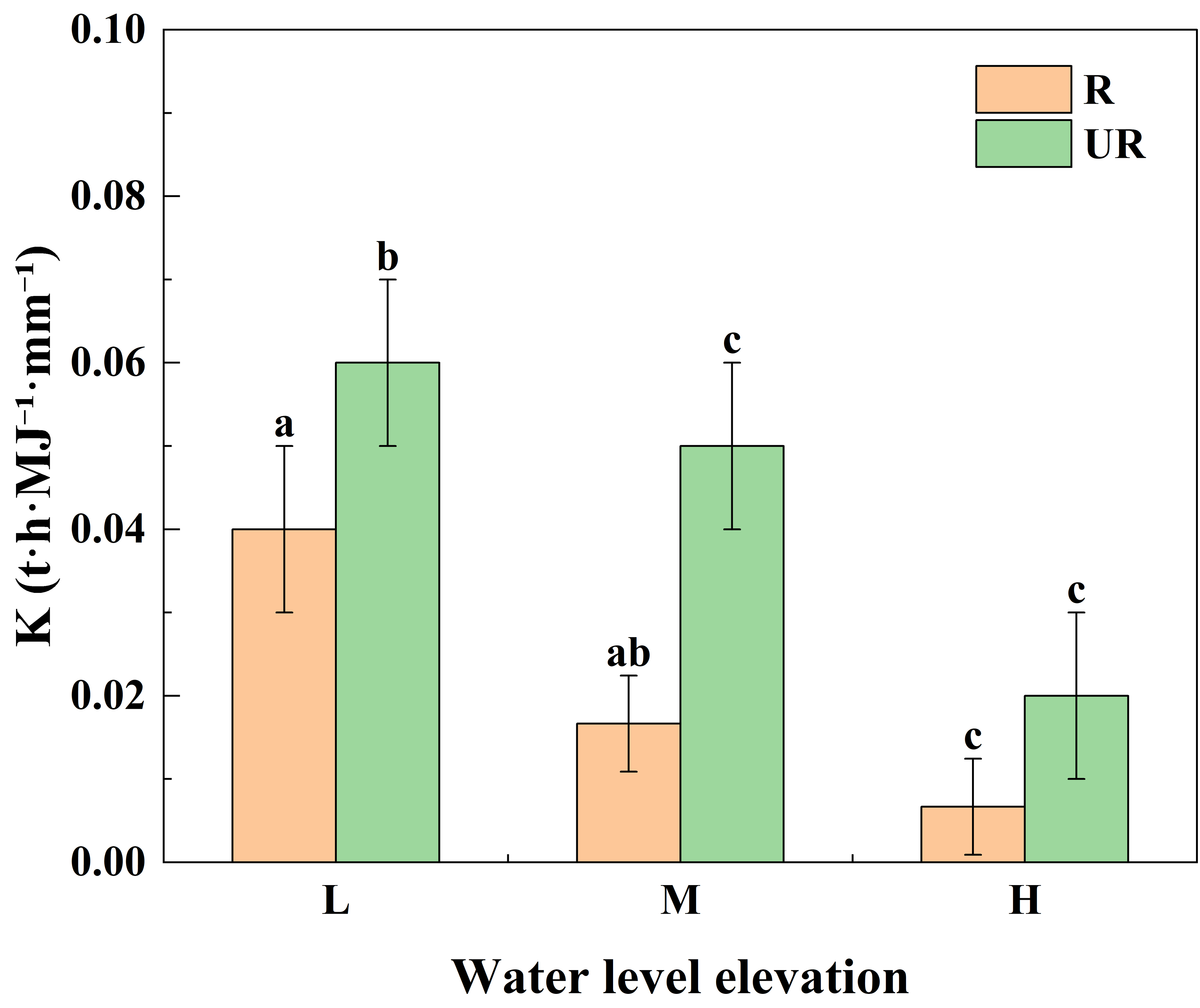

Supplement: S1 File — (ZIP) [file pone.0336637.s001.zip › S1/Fig 7.tif]

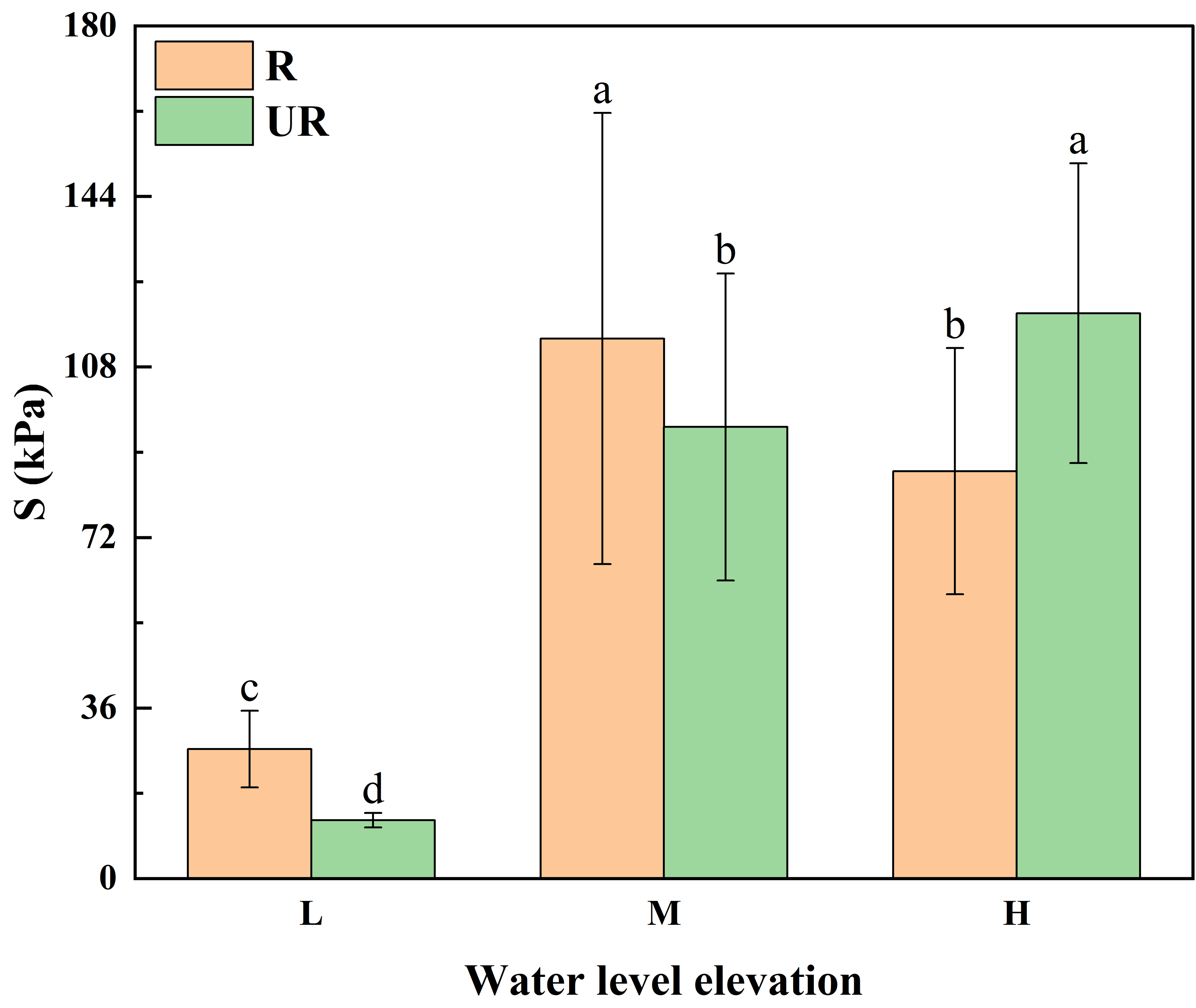

Supplement: S1 File — (ZIP) [file pone.0336637.s001.zip › S1/Fig 8.tif]

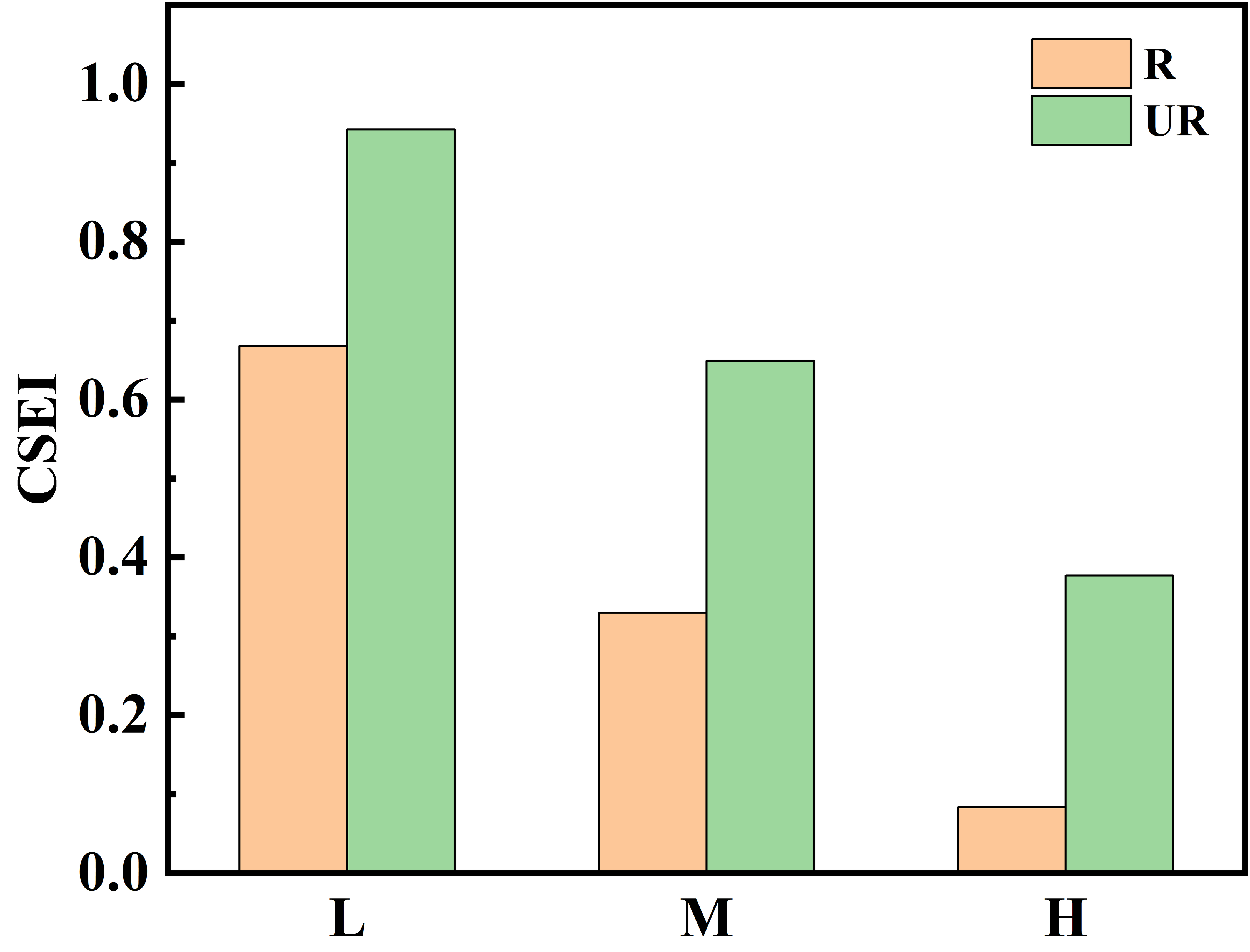

Supplement: S1 File — (ZIP) [file pone.0336637.s001.zip › S1/Fig 9.tif]

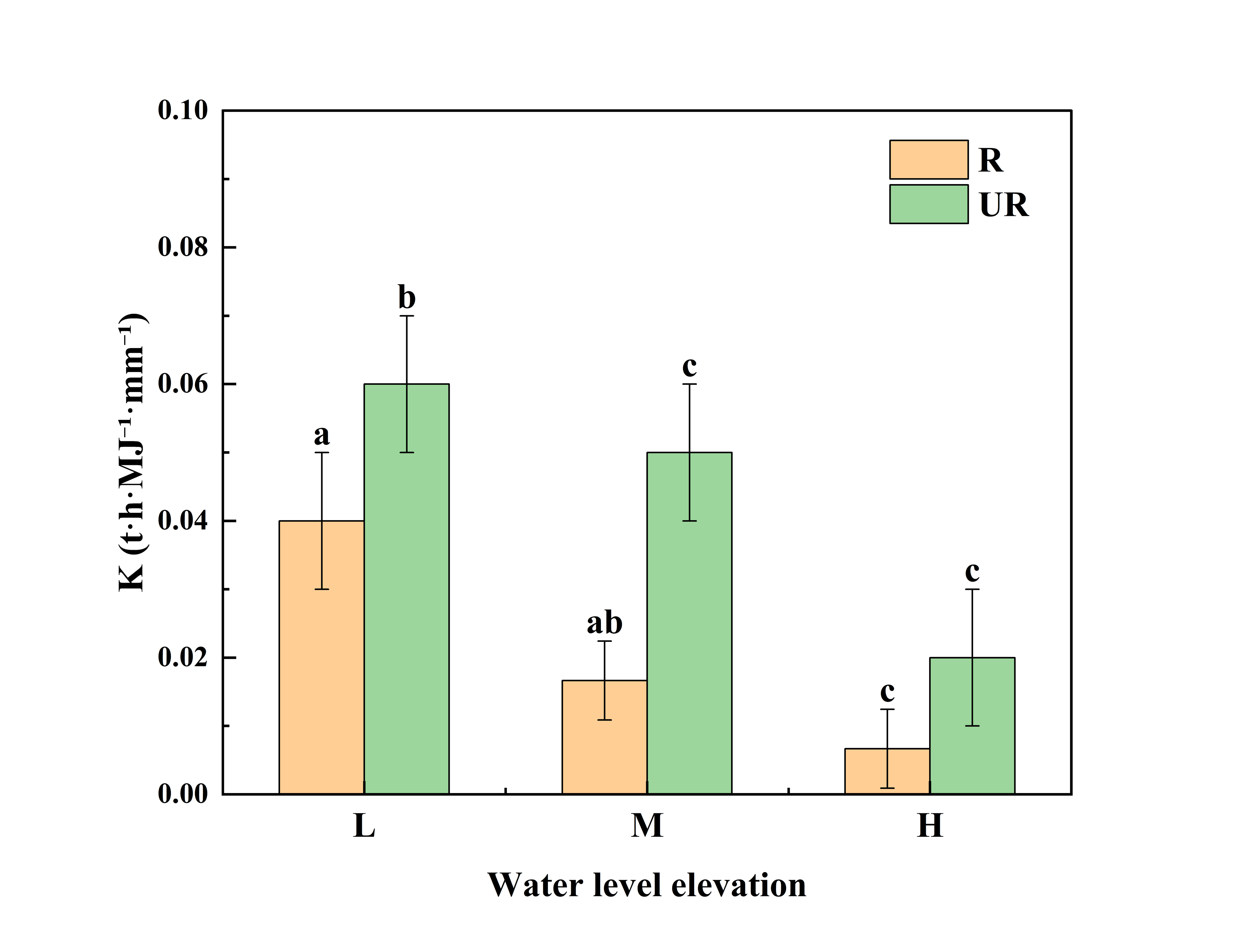

Supplement: S1 File — (ZIP) [file pone.0336637.s001.zip › S1/K.tif]

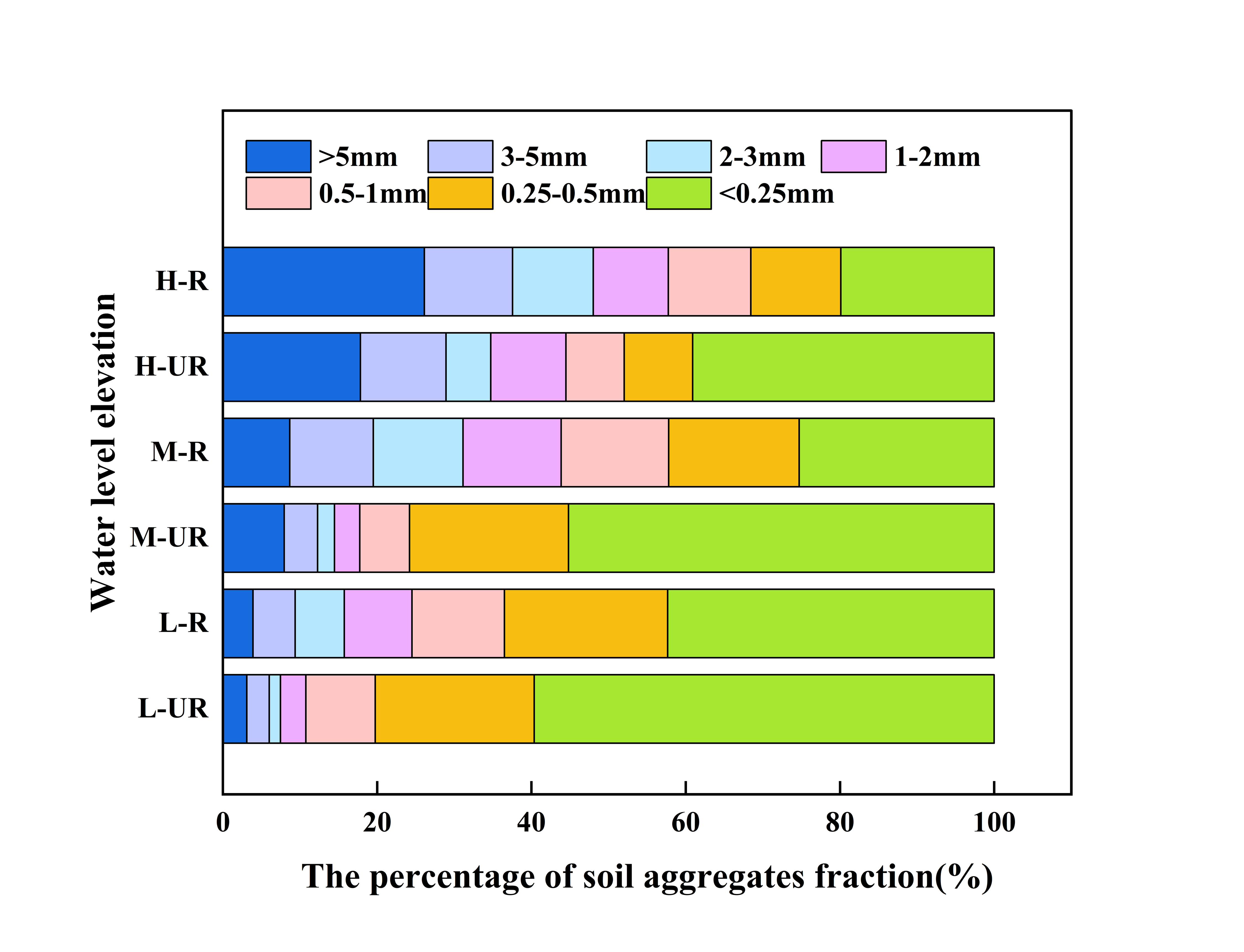

Supplement: S1 File — (ZIP) [file pone.0336637.s001.zip › S1/lijing.tif]

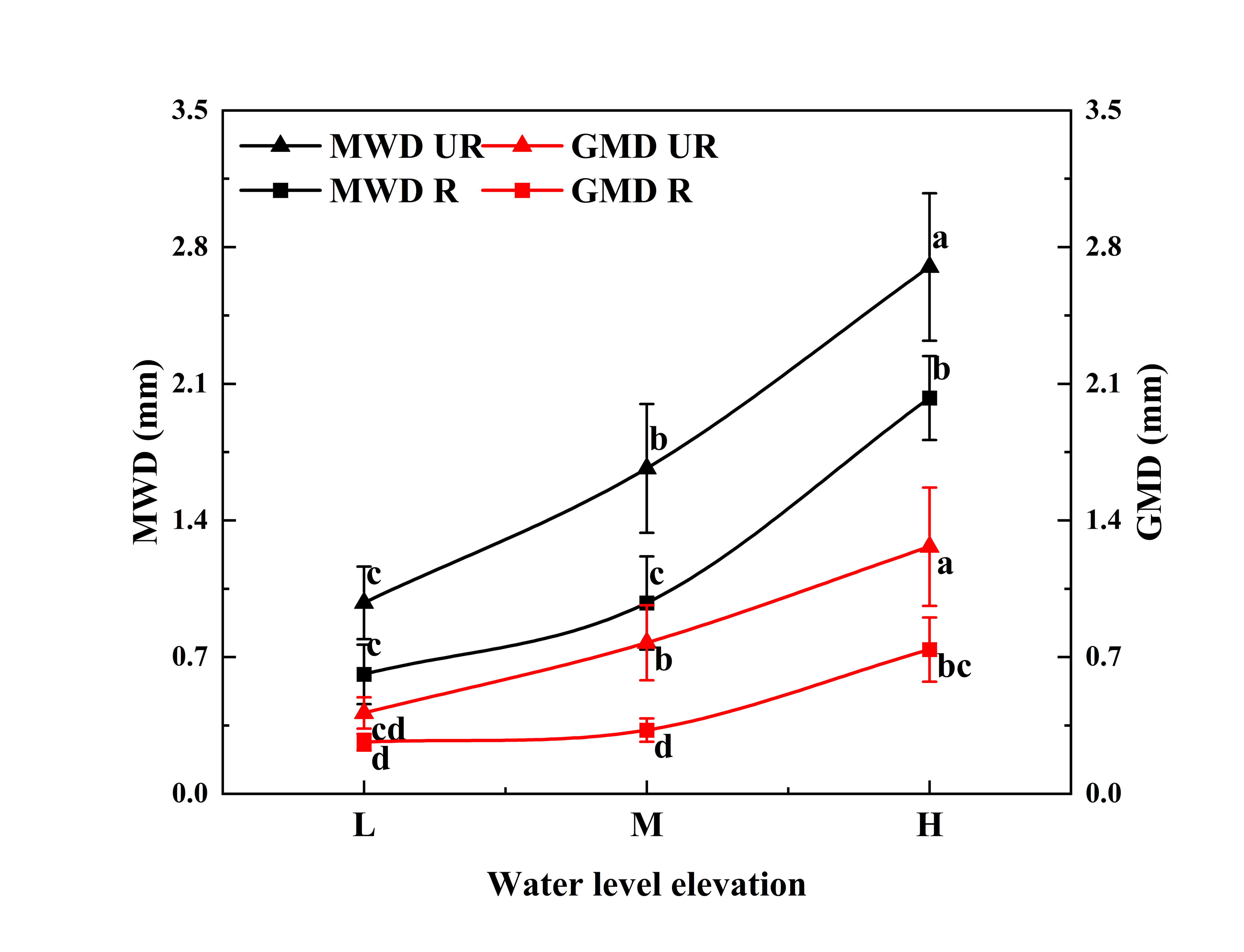

Supplement: S1 File — (ZIP) [file pone.0336637.s001.zip › S1/mwd gmd.tif]

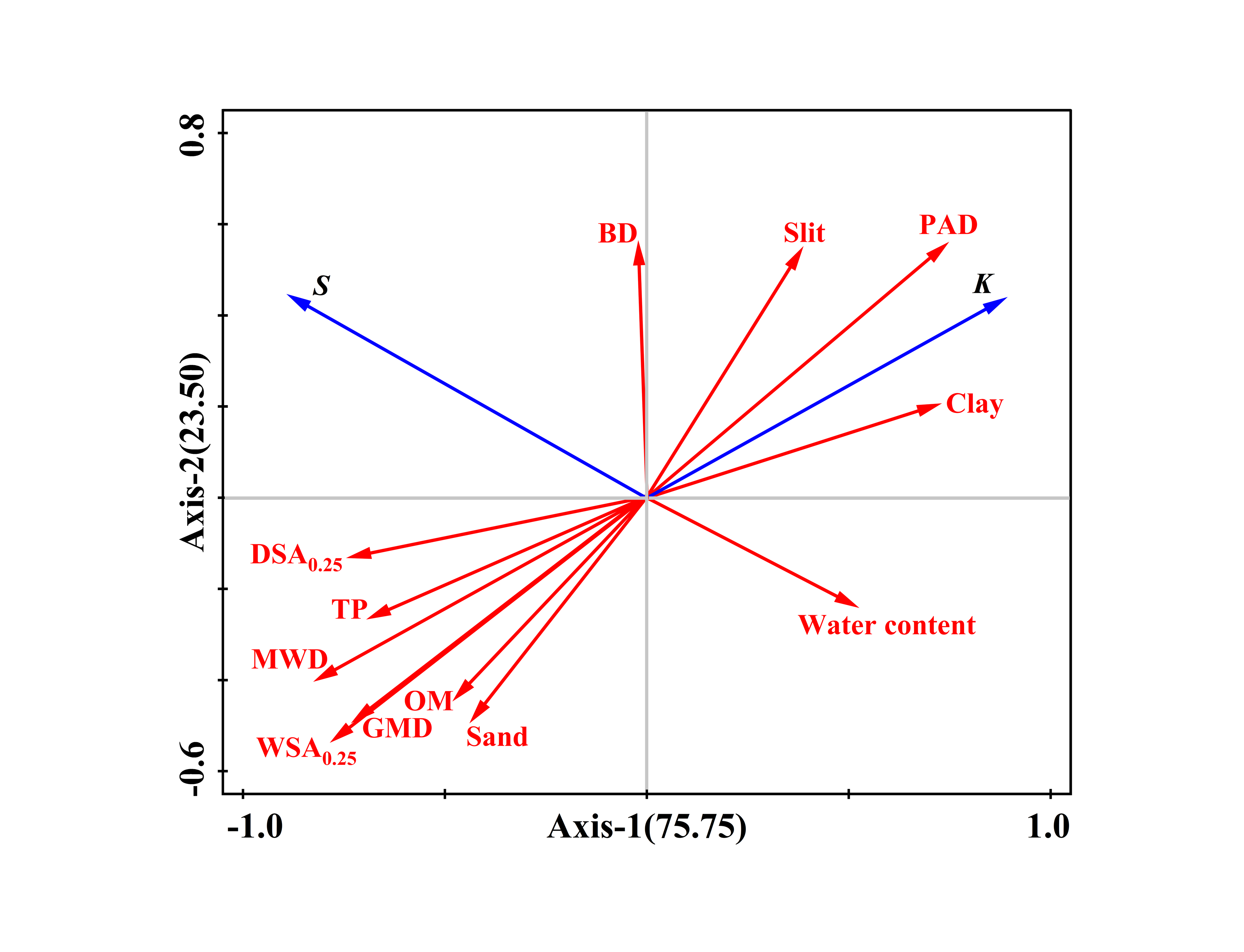

Supplement: S1 File — (ZIP) [file pone.0336637.s001.zip › S1/RDA.tif]

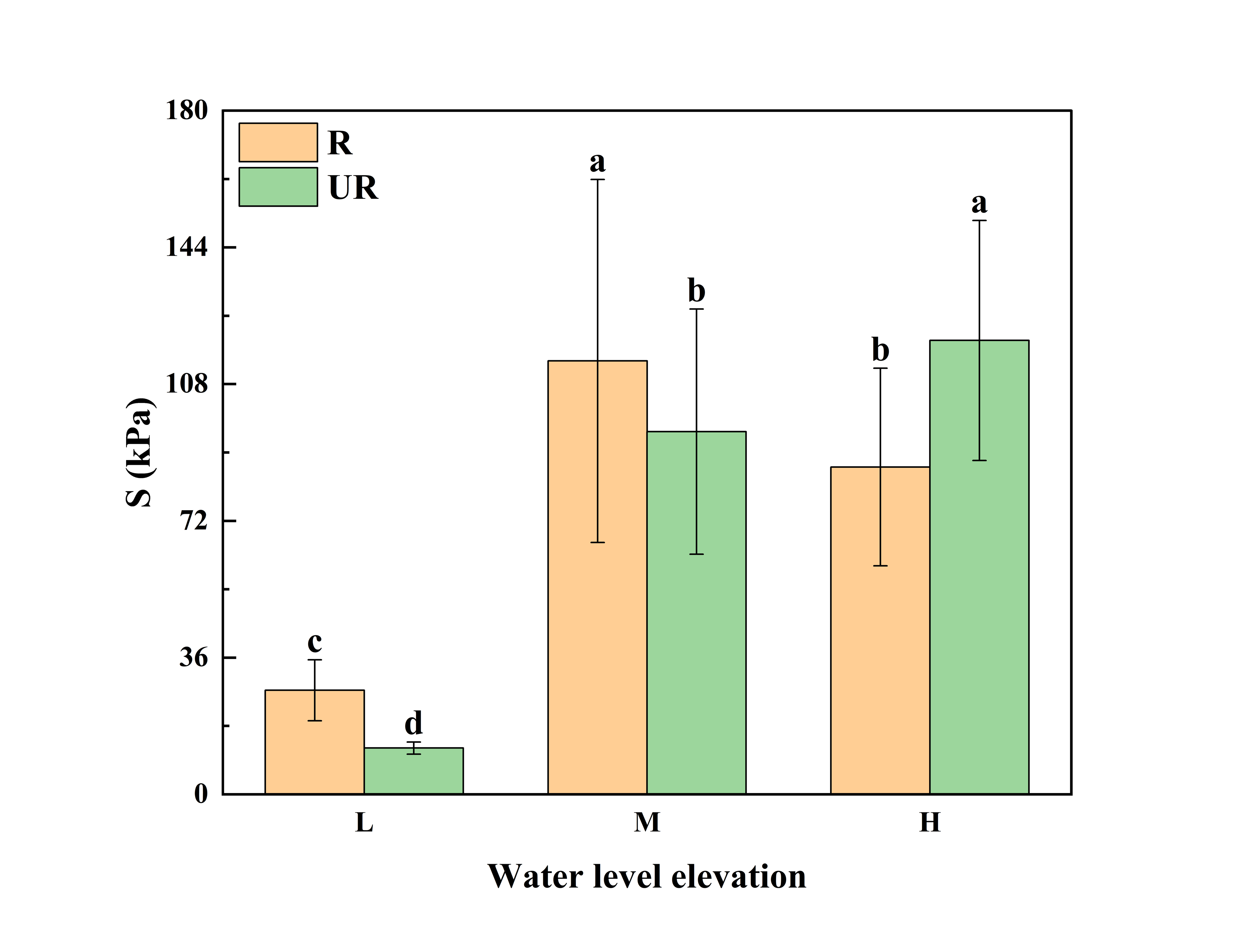

Supplement: S1 File — (ZIP) [file pone.0336637.s001.zip › S1/s.tif]
