# Supplementary material for: Quantitative evaluation of soil anti-erodibility in the fluctuation zones of rooted soil in a large reservoir, southwest of China
Source: PLoS One. 2026 Mar 10;21(3):e0336637. doi: 10.1371/journal.pone.0336637 (PMC12974866; doi:10.1371/journal.pone.0336637)
Supplement: Attachment — Submitted filename: review.docx [file pone.0336637.s002.docx]

**I. Structural Issues**

- **Abstract Section**
  - **Issue:** The description of results in the Abstract (e.g., specific numerical ranges and comparisons of the CSEI index) is overly detailed, exceeding the requirement for conciseness. This risks obscuring the key points.
  - **Suggested Revision:** Simplify specific data points; focus on core conclusions (e.g., "CSEI decreases with increasing elevation"). Emphasize key trends rather than precise numerical values.
- **Introduction Section**
  - **Issue:** The discussion on the role of plant roots (e.g., "root traits may mediate the impact of plant diversity") lacks a tight logical connection to the subsequent research objectives. It fails to explicitly link root systems with the interactive effects of water level fluctuations.
  - **Suggested Revision:** Supplement the explanation with the unique mechanisms of root systems in water fluctuation zones, such as root adaptations to wet-dry cycles and their regulatory role on soil anti-erodibility.
- **Discussion Section**
  - **Issue:** There is content overlap between Section 4.1 (Impact of Water Level Changes on Soil Physicochemical Properties) and Section 4.2 (Impact of Soil Properties on Erosion). For example, the statement "Soil organic matter content increases with elevation" appears in both sections, creating logical redundancy.
  - **Suggested Revision:** Integrate overlapping content. Reorganize paragraphs according to the logical chain: "Water Level Fluctuations → Changes in Soil Properties → Anti-Erodibility Response".
- **Conclusion Section**
  - **Issue:** In Conclusion 1, the description of "root zone soil K value variation with elevation" (e.g., "High (0.007) < Medium (0.017) < Low (0.038)") omits units (e.g., "t·h·MJ⁻¹·mm⁻¹"), violating academic standards.
  - **Suggested Revision:** Add the units for the K value and ensure consistent data presentation format throughout the paper.

**II. Academic Quality Issues**

- **Limitations of Research Methods**
  - **Issue:** In Section 2.4 (Data Analysis Methods), Principal Component Analysis (PCA) reduced 14 indicators to 3 principal components, but the rationale for selecting the first 3 components (e.g., eigenvalue >1 criterion) is not provided. Reliability tests for the PCA (e.g., KMO test or Bartlett's test of sphericity) are also missing.
  - **Suggested Revision:** Supplement the results of PCA suitability tests (KMO, Bartlett's) and justify the cumulative variance contribution rate (85.143%).
- **Insufficient Depth in Data Discussion**
  - **Issue:** The analysis of soil shear strength variation with elevation in Section 3.2.3 (e.g., "root zone soil shear strength decreases at high elevation") lacks integration with interacting factors like root density or soil moisture content, making the explanation inadequate.
  - **Suggested Revision:** Correlate with root biomass data (e.g., high elevation vegetation coverage 78%). Analyze that the potential negative correlation between root density and shear strength might be caused by increased soil moisture leading to soil softening.
- **Timeliness and Relevance of References**
  - **Issue:** Reference [16] (Wischmeier, 1978) is a classic model, but recent advances in the field (e.g., studies on soil erosion in reservoir fluctuation zones post-2020) are not cited. Some references (e.g., [65]) are cited redundantly.
  - **Suggested Revision:** Supplement relevant studies from 2020-2025. Replace redundant citations to enhance the study's currency.

**III. Grammatical Issues**

- **Mixed Chinese-English Formatting and Errors**
  - **Issue:** Formula symbols like "DSA 0.25" are inconsistently mixed with Chinese text in the Abstract. Some paragraphs (e.g., paragraph 3 of the Abstract) contain grammatical errors, such as the misspelling "t is sisnificntly positively correlated".
  - **Suggested Revision:** Standardize formula symbol formatting (e.g., use "DSA₀.₂₅"). Correct spelling errors to "it is significantly positively correlated".
- **Inconsistent Terminology**
  - **Issue:** Terms like "colluvial slope soils" (Abstract) and "colluvial zone soil" (Abstract) are used inconsistently. "Erosion coefficient" and "erodibility factor K" are used interchangeably, causing confusion.
  - **Suggested Revision:** Standardize terms (e.g., use "colluvial zone soil" and "erodibility factor K" consistently). Define full terms upon first appearance.
- **Wordy Sentences and Logical Gaps**
  - **Issue:** The last paragraph of the Introduction ("Over all the response of soil detachment...") has overly complex sentence structure and grammatical gaps (e.g., missing subject before "are in need of further study").
  - **Suggested Revision:** Break into shorter sentences, e.g.: "However, the quantitative effects of these factors and their dominant influences remain unknown. It is speculated that root traits may mediate...".

**IV. Errors in Methods, Knowledge, and Techniques**

- **Formula Citation and Derivation Issues**
  - **Issue:** In Section 2.2, the calculation formulas (1) and (2) for the soil erodibility factor K are cited from Shirazi et al., 1988, but the specific improvements made by Zhang et al., 2008 (e.g., parameter adjustments or applicability extensions) are not explained. Furthermore, "logGMD" in formula (1) lacks specification of the logarithm base (should be natural logarithm ln).
  - **Suggested Revision:** Supplement the specific steps of the improvement method. Correct the formula to "ln GMD" and clearly cite the source.
- **Insufficient Rigor in Statistical Analysis**
  - **Issue:** In Section 3.1, the statement "root zone soil bulk density decreased by 24.39% compared to non-root zone" lacks indication of statistical significance (e.g., p-value from t-test or ANOVA). Merely describing numerical differences does not meet academic standards.
  - **Suggested Revision:** Supplement significance test results, e.g., "(p < 0.05, t-test)", and add statistical symbols (e.g., asterisks) to Figure 3.
- **Ambiguous Definition of Technical Indicators**
  - **Issue:** In Section 2.3, the definition of "PAD (X12)" as "aggregate of destruction" is unclear regarding its relationship to "aggregate destruction rate". Additionally, units for "DSA 0.25" and "WSA₀.₂₅" in formula (4) are missing (should be mass percentage).
  - **Suggested Revision:** Clarify that PAD refers to the ">0.25 mm aggregate destruction rate". Supplement the units and physical meaning of all parameters in the formulas.

**V. Comprehensive Revision Suggestions**

- **Structural Optimization:** Reorganize the Discussion section logic to avoid repetition. Supplement data units and trend analysis in the Conclusions.
- **Academic Rigor:** Enhance PCA validation procedures, supplement supporting data (e.g., root biomass), update references.
- **Language Standardization:** Unify terminology, correct grammatical errors, optimize formula symbol formatting.
- **Methodological Refinement:** Elaborate on formula improvement details, add statistical significance tests, standardize indicator definitions.
