# Supplementary material for: Quantitative evaluation of soil anti-erodibility in the fluctuation zones of rooted soil in a large reservoir, southwest of China
Source: PLoS One. 2026 Mar 10;21(3):e0336637. doi: 10.1371/journal.pone.0336637 (PMC12974866; doi:10.1371/journal.pone.0336637)
Supplement: Attachment — Submitted filename: Response to the Reviewers.docx [file pone.0336637.s004.docx]

**Response to the Reviewers**

**Dear Editors and Reviewers:**

Thank you for your letter and for the reviewers’ comments concerning our manuscript entitled “Quantitative evaluation of soil anti-erodibility in the fluctuation zones of rooted soil in a large reservoir, southwest of China” (Manuscript ID: PONE-D-25-21716). Those comments are all valuable and very helpful for revising and improving our paper, as well as the important guiding significance to our research. We have studied comments carefully and made corrections which we hope to meet with approval. Revised portions are marked in the paper. The responds to the reviewer’s comments are as follows:

**The information of the corresponding author's institution has changed：**

Due to personnel changes, the corresponding author Xiao Henglin's workplace was changed to State Key Laboratory of Precision Blasting, Jianghan University, Wuhan, 430056, China.

**Affiliated institution changed to:**

1. Hubei Key Laboratory of Environmental Geotechnology and Ecological Remediation for Lake & River, Hubei University of Technology, Wuhan 430068, China.

2. Key Laboratory of Intelligent Health Perception and Ecological Restoration of Rivers and Lakes, Ministry of Education, Hubei University of Technology, Wuhan 430068, China.

3. State Key Laboratory of Precision Blasting, Jianghan University, Wuhan, 430056, China

**Changed in the Acknowledgments to:**

This research was supported by the National Natural Science Foundation of China (No. 42307256), the Joint Funds of the Nature Science Foundation of Hubei Province (No. 2022CFD172), the Joint Funds of the National Nature Science Foundation of China (U22A20232), the Innovation Research Group Project of the Hubei Provincial Department of Science and Technology (2025AFA020), the Open Project Funding of Hubei Key Laboratory of Environmental Geotechnology and Ecological Remediation for Lake & River (HJKFYB202405), the Innovation Research Team Project of the Hubei Provincial Department of Science (No.T2024006) and the Innovation Demonstration Base of Ecological Environment Geotechnical, Ecological Restoration of Rivers and Lakes (2020EJB004).

**The second author of the article has been changed from Zexi Song to Xinghan Niu, and Zexi Song has been moved to the fifth author. Please note this change.**

**Comments and Suggestions for Authors**

I. **Structural Issues**

**•	Abstract Section**

Q1: **Issue**: The description of results in the Abstract (e.g., specific numerical ranges and comparisons of the CSEI index) is overly detailed, exceeding the requirement for conciseness. This risks obscuring the key points.

R1：We sincerely appreciate the reviewer’s insightful feedback regarding the level of detail in the Abstract. We agree that excessive numerical specifics may detract from the core conclusions and compromise conciseness. Accordingly, we have comprehensively revised the Abstract to prioritize clarity and emphasize key trends, as suggested.

In the abstract section, I have removed the specific numerical values and correlation coefficients of CSEI, such as "correlation coefficients of -0.70, -0.5, and -0.52, respectively," and detailed figures like "High (root zone soil) 0.082 < Middle (root zone soil) 0.330 < High (un-rooted zone soil) 0.377 < Medium (un-rooted zone soil) 0.649 < Low (root zone soil) 0.668 < Low (un-rooted zone soil) 0.942." The focus was kept on the key trends and core conclusions to maintain the abstract's conciseness.

**••	Introduction Section**

Q2: Issue: The discussion on the role of plant roots (e.g., "root traits may mediate the impact of plant diversity") lacks a tight logical connection to the subsequent research objectives. It fails to explicitly link root systems with the interactive effects of water level fluctuations.

R2：We thank the reviewer for this valuable observation. We agree that a clearer mechanistic link between root systems, water-level fluctuations, and soil anti-erodibility was needed to strengthen the logical flow towards our research objectives.

In lines 141-152 of the article, I added content related to how plant roots adapt to environmental changes under alternating dry and wet conditions by altering their morphological structure, thereby affecting erosion resistance, and established a link between the interaction of roots and water level fluctuations.From: "In addition, the effects of plant roots on soil properties also differ with respect to various root characteristics and environmental conditions. Under alternating dry and wet conditions, plant root systems adapt to environmental changes by altering their morphological structure." up to the end of this paragraph is the new content I supplemented. Additionally, I added a paragraph in lines 70-77 of the introduction section to briefly explain what soil erosion is, making the introduction more comprehensive.

**•	Discussion Section**

Q3:Section 4.1 (Impact of Water Level Fluctuations on Soil Physicochemical Properties) and Section 4.2 (Impact of Soil Properties on Erosion) exhibit content overlap.

R3：Thank you for your valuable feedback. As suggested, we have restructured the relevant paragraphs according to the logical framework of "water level fluctuations → changes in soil properties → anti-erosion response." To improve clarity and focus, all content pertaining to anti-erosion effects has been consolidated into Section 4.2, while Section 4.1 now exclusively discusses the influence of water level changes on soil physicochemical properties, without reference to erosion-related aspects. Some overlapping content may remain, and we would be grateful for any further suggestions you may have.

**•	Conclusion Section**

Q4: In Conclusion 1, the description of root zone soil potassium values varying with elevation (e.g., 'high (0.007) < medium (0.017) < low (0.038)') omits the units, violating academic standards.

R4：Thank you for your valuable feedback. We have addressed the points you raised in the corresponding sections. Specifically, the unit of K ("t·h·MJ⁻¹·mm⁻¹") has now been added to Conclusion 1, as well as to formula (2) in Section 2.2.

II. **Academic Quality Issues**

Q5: In Section 2.4 (Data Analysis Methods), Principal Component analysis (PCA) reduces 14 indicators to 3 principal components, but does not provide reasons for choosing the first 3 components (for example, eigenvalue >1 criterion). The reliability tests of PCA (such as KMO test or Bartlett sphericity test) are also missing.

R5：Thank you for your valuable feedback. We have incorporated the KMO test and Bartlett’s test of sphericity into the manuscript. Furthermore, based on the scree plot, the first three principal components were retained, as presented below：

| KMO Sampling Adequacy Measure | | 0.647 |
| --- | --- | --- |
| Bartlett's test of sphericity | Approximate chi square | 332.68 |
|  | freedom | 66 |
|  | significance | 0 |

Q6: The analysis of soil shear strength variation with elevation in Section 3.2.3 lacks integration with interacting factors like root density or soil moisture content, making the explanation inadequate.

R6：Thank you for your valuable feedback. Due to the fact that our sampling was focused exclusively on root-related data, we have incorporated corresponding data on changes in plant root systems in relation to water level variations into the concluding segment of the second paragraph in Section 2.2.

| Elevation | length (cm) | surface area (cm^2^) | mean root diamete（mm） | Total Root Volume（cm^3^） |
| --- | --- | --- | --- | --- |
| Low | 17.1920±3.7470c | 1.9814±0.5618c | 0.3607±0.0789b | 0.0212±0.0039b |
| Mid | 20.5585±3.4703b | 3.3160±0.5553b | 0.4111±0.0622ab | 0.0455±0.0076a |
| High | 26.8330±5.1689a | 3.9784±0.2801a | 0.4775±0.0640a | 0.0470±0.0023a |

In response, we expanded on the potential reasons for the negative correlation between root density and shear strength, as shown in Section 4.2, lines 603-613.

Q7：Reference [16] (Wischmeier, 1978) is a classic model, but recent advances in the field (e.g., studies on soil erosion in reservoir fluctuation zones post-2020) are not cited. Some references (e.g., [65]) are cited redundantly.

R7：Thank you for your valuable feedback. In response to your suggestions, we have revised the introduction to include recent advancements in the field. Specifically, we have incorporated relevant studies by Kinnell (2015), Thomas and Joseph et al. (2018), and Duan and Bai et al. (2020) into the second paragraph of the introduction to enhance the timeliness and relevance of our literature review (Kinnell, 2015; Thomas and Joseph et al., 2018; Duan and Bai et al., 2020). Additionally, we have streamlined the reference list by removing redundant citations. However, a small number of seminal but older references have been retained due to their foundational role in the topic and the current lack of modern alternatives.

III. **Grammatical Issues**

Q8: Formula symbols like "DSA 0.25" are inconsistently mixed with Chinese text in the Abstract. Some paragraphs (e.g., paragraph 3 of the Abstract) contain grammatical errors, such as the misspelling "t is sisnificntly positively correlated".

R8: Thank you for your valuable feedback. We have revised the manuscript accordingly. Specifically, the notation "DSA 0.25" has been standardized to "DSA₀.₂₅" using proper subscript formatting. Additionally, a spelling error has been corrected in the sentence previously stating "t is sisnificntly positively correlated", which now reads "it is significantly positively correlated."

Q9: Terms like "colluvial slope soils" (Abstract) and "colluvial zone soil" (Abstract) are used inconsistently. "Erosion coefficient" and "erodibility factor K" are used interchangeably, causing confusion.

R9: Thank you for your valuable feedback. In accordance with your suggestions, we have carefully revised the manuscript to ensure terminological consistency. Specifically, the terms "colluvial zone soil" and "erodibility factor K" have been standardized and used consistently throughout the article.

Q10:The last paragraph of the Introduction ("Over all the response of soil detachment...") has overly complex sentence structure and grammatical gaps (e.g., missing subject before "are in need of further study")

R10: Thank you for your valuable feedback. We have carefully revised the manuscript accordingly. Specifically, the original sentence “Over all the response of soil detachment...” has been restructured into two clearer sentences: “The response of soil detachment to different combinations of soil and root system properties has not been fully quantified. More research is needed in this area.” Additionally, the sentence “it was speculated that...” has been corrected to “It is speculated that...” to maintain appropriate academic tone and grammatical consistency.

IV. **Errors in Methods, Knowledge, and Techniques**

Q11: The sentence "Over all the response of soil detachment..." was changed into two sentences: "The response of soil detachment to different combinations of soil and root system properties has not been fully quantified. More research is needed in this area." Additionally, the phrase "it was speculated that root traits may mediate the impact of plant diversity on riparian soil stability." was revised to "It is speculated that root traits may mediate the impact of plant diversity on riparian soil stability."

R11: Thank you very much for your thoughtful comments regarding the methodological considerations in our study.

In response to your feedback, we would like to clarify the rationale behind the selection of the K factor formula. As noted by Zhang et al. (2008), the direct application of the three original K factor estimation methods under Chinese conditions resulted in notable overestimations. Accordingly, Zhang and colleagues recalibrated the formula to improve its suitability for regional soil conditions, and it is this revised formula that has been adopted in our study.

Regarding the logarithmic base used in the formula, we acknowledge that Shirazi et al. (1988) 【Shirazi, M. A. and L. Boersma, et al. (1988). "A unifying quantitative analysis of soil texture: improvement of precision and extension of scale." Soil Science Society of America Journal 52 (1): 181-190.】employed base 10 in their original work. However, subsequent applications—including recent studies such as Wu et al. (2023)—have consistently utilized the natural logarithm 【log(GMD)】 in related contexts. 【Wu, T. and Y. Zhang, et al. (2023). "Factors affecting the stability of soil aggregates of plinthosols in the middle reaches of the Yangtze River." Catena 228: 107159.】In order to maintain consistency with prevailing literature and ensure broader comparability of results, we retained the natural logarithm in our formula. A clarifying note has been included beneath the equation to explicitly indicate the use of base 10 where relevant, thereby preserving both mathematical integrity and explanatory clarity.

Q12: In Section 3.1, the statement "root zone soil bulk density decreased by 24.39% compared to non-root zone" lacks indication of statistical significance (e.g., p-value from t-test or ANOVA). Merely describing numerical differences does not meet academic standards.

R12: Thank you for your valuable feedback. In response to your suggestions, we have added descriptions of significant differences throughout the Results section. For instance, the following statement has been included: “At each elevation, the bulk density of the root soil was significantly lower than that of the rootless soil (P < 0.05) at both low and high elevations, with reductions of 24.39% and 24.86%, respectively.”

Additionally, we have included asterisks (*) in Figure 5.

Q13: In Section 2.3, the definition of "PAD (X12)" as "aggregate of destruction" is unclear regarding its relationship to "aggregate destruction rate". Additionally, units for "DSA 0.25" and "WSA₀.₂₅" in formula (4) are missing (should be mass percentage).

R13: Thank you for your valuable feedback.In response, we have clarified the terminology and units throughout the manuscript. Specifically, the term PAD is now consistently defined as "aggregate destruction rate" in all relevant table captions and equation descriptions. Additionally, units have been explicitly added for DSA₀.₂₅ and WSA₀.₂₅ as ($wt\%$) wherever applicable.

V. Comprehensive Revision Suggestions

Thank you very much for your thorough review and valuable suggestions. We have carefully revised the manuscript accordingly to address your comments. The main improvements are summarized as follows:

In the Discussion section, the content has been reorganized along the logical chain of “water level fluctuations → changes in soil properties → anti-scour response” to improve clarity and avoid redundancy. Units have been consistently added for key parameters: K as (t·h·MJ⁻¹·mm⁻¹), DSA₀.₂₅ as (wt%), and WSA₀.₂₅ as (wt%).

In Section 2.4, the KMO test and Bartlett’s test of sphericity have been included to strengthen the validation of the Principal Component Analysis (PCA). Additionally, root-related data have been supplemented where appropriate.

In the second paragraph of the Introduction, we have incorporated the most recent research advances in the field and removed the majority of redundant references. However, a limited number of classical references were retained due to the unavailability of contemporary alternatives for foundational models.

Terminology has been standardized throughout the manuscript—e.g., “DSA₀.₂₅”, “soil erodibility factor K”, and “colluvial zone soil”—and grammatical errors have been corrected. Furthermore, significance testing statements have been added where applicable, and PAD is now explicitly defined as "aggregate destruction rate" in all relevant sections.

We believe these revisions have significantly improved the quality and clarity of the manuscript and thank you once again for your constructive input.

**Special thanks to you for your good comments.**

We have made every effort to improve the manuscript and have implemented some changes within it. These alterations are designed to enhance the clarity and presentation without affecting the core content and structure of the paper. Instead of listing the changes here, we have highlighted them in the revised manuscript for your direct reference.

We extend our sincere gratitude to the Editors and Reviewers for their diligent work and constructive feedback. We hope that these revisions meet with your approval. Once again, thank you very much for your valuable comments and suggestions.

With best wishes,

Yours sincerely,

Henglin Xiao & Pengcheng Wang
